# Acute promyelocytic leukemia presenting as recurrent spinal myeloid sarcomas 3 years before developing leukemia: A case report with review of literature

**DOI:** 10.1002/ccr3.1991

**Published:** 2019-01-08

**Authors:** Tomoko Yamashita, Akihiko Nishijima, Yuma Noguchi, Kensuke Narukawa, Gaku Oshikawa, Hina Takano

**Affiliations:** ^1^ Department of Internal Medicine Toshiba General Hospital Tokyo Japan; ^2^ Department of Hematology Japan Red Cross Musashino Hospital Tokyo Japan; ^3^ Division of Blood Transfusion Japan Red Cross Musashino Hospital Tokyo Japan

**Keywords:** acute promyelocytic leukemia, fluorescence in situ hybridization, myeloid sarcoma, spinal tumor

## Abstract

The de novo myeloid sarcoma (MS) type of acute promyelocytic leukemia (APL) is rare, and clinical features may differ from extramedullary diseases in advanced APL. Many cases occur as a spinal tumor, and some occur in the absence of bone‐marrow diseases or coagulation abnormalities. Fluorescence in situ hybridization analysis of MS tissue is useful for accurate diagnosis, even in preserved tissue.

## 1 INTRODUCTION

Fifty‐year‐old man presented with paralysis caused by a vertebral body tumor. The tumor was a myeloid sarcoma (MS) without signs of leukemia. Chemotherapy and irradiation resulted in short remission. Acute promyelocytic leukemia (APL) became obvious during the second relapse. Fluorescence in situ hybridization (FISH) analysis of preserved MS tissue indicated de novo MS/APL.

Myeloid sarcoma is a tumor mass consisting of myeloid blasts with or without maturation and occurs in sites other than the bone marrow. It is often described as an extramedullary disease (EMD) developing in patients with acute myeloid leukemia (AML).^1^ In particular, MS without any history of leukemia, myelodysplastic syndrome, or myeloproliferative neoplasm is defined as de novo MS. Specific types of AML, such as myelomonocytic leukemia and monocytic leukemia, tend to develop MS/EMD more than other types.

In APL, approximately 3%‐5% have complications of MS/EMD and are usually concurrent with disease relapse.^2^, ^3^, ^4^ In contrast, de novo MS as the initial manifestation of APL occurs in <10% of EMD cases. Little is known about the clinical profile and treatment options for this rare type of disease, which may differ from EMD’s developing in a relapse phase.

Here, we present a case of recurrent de novo MS in the spine. Initially, there was no sign of leukemia in the bone marrow or peripheral blood and no coagulation abnormality. Signs of APL became recognizable only after transforming into leukemia 3 years from initial onset. Although there are several reports of the de novo MS/APL lacking bone‐marrow invasion at their onset, we believe this is the longest latent period before the development of bone‐marrow disease. Retrospective analysis of the preserved initial MS‐tissue sample revealed PML/RARα fusion gene by FISH, conforming the diagnosis of the de novo MS type of APL.

## 2 CASE REPORT

**Figure 1:**
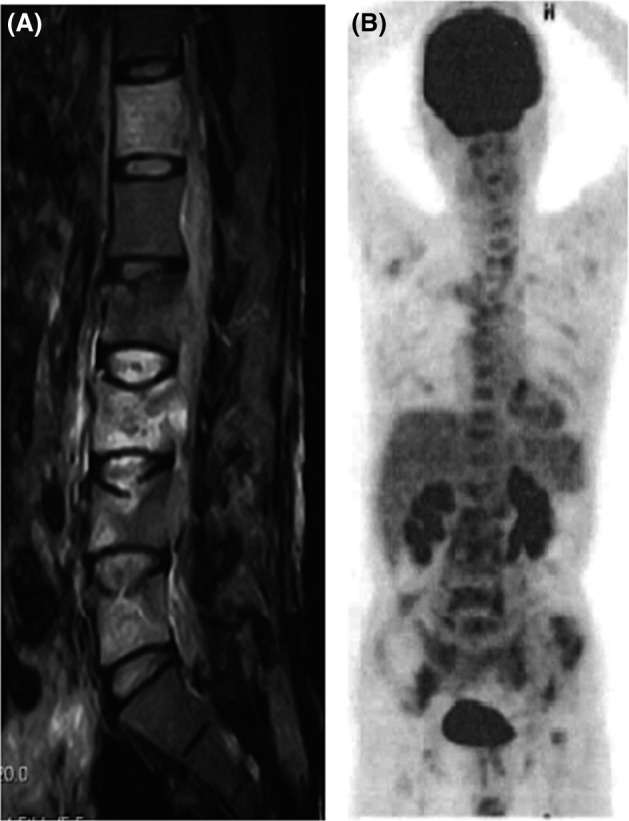
A, Sagittal T2‐weighted magnetic resonance imaging of the spine. A tumor emerging from the vertebral bodies of L2 through L4 is present. In addition, high signal intensity in the vertebral bodies of Th12, L3‐L5 is evident. B, 18F‐fluoro‐deoxy‐glucose positron‐emission tomography image showing nodular uptakes in the vertebras, ribs, pelvis, and femur. Standardized uptake value given as the maximum pixel value in the tumor was 3.54 at the right pelvis, where it was increased the most

A 50‐year‐old Japanese man presented to our hospital complaining of numbness and paralysis of the left foot. Magnetic resonance imaging showed a tumor mass around the vertebral bodies, which was invading the spinal canal from L2 through L4 (Figure 1A). The tumor originated from the posterior wall of the lumbar vertebrae and was compressing the dura mater. In addition, there were multiple abnormal signals within the T12, L3‐5 vertebral bodies.

**Figure 2:**
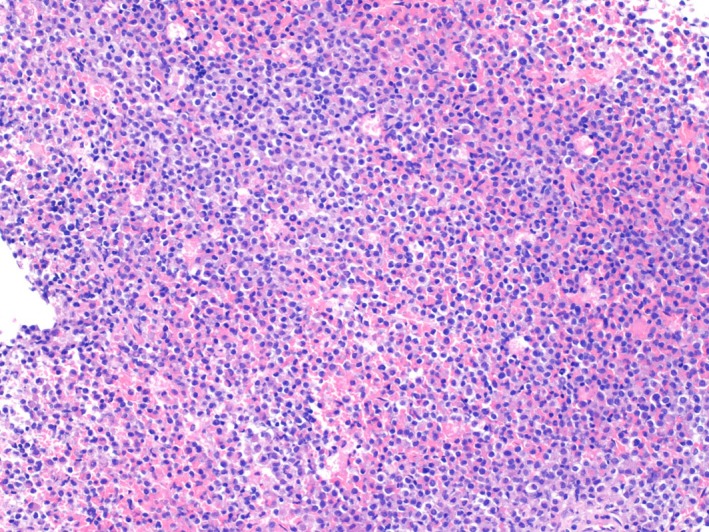
Hematoxylin‐eosin–stained section of the vertebral mass. Tumor cells with eosinophilic cytoplasm are diffusely infiltrating the bone tissue (original magnification ×100)

Systemic examination by 18F‐fluoro‐deoxy‐glucose (FDG) positron‐emission tomography/computed tomography (PET/CT) showed multiple nodular FDG uptakes in the vertebrae, ribs, pelvis, and femur (Figure 1B). Needle biopsies of the L5 vertebra showed no sign of tumor cells, and the cerebrospinal‐fluid examinations were normal. Finally, partial excision of the tumor mass by surgical procedure was performed for diagnosis. Microscopic examination revealed mononuclear tumor cells with eosinophilic cytoplasm infiltrating between the bone trabeculae (Figure 2). The tumor cells were positive for CD33 and CD68 and negative for CD3, CD20, CD34, and CD56, which confirmed the diagnosis of MS.

Laboratory tests showed no abnormalities in blood count and coagulation tests. There was no sign of leukemia morphologically in the bone marrow. Cytogenetic examination revealed 46, XY and was negative for translocation of PML/RARα and other balanced translocations routinely searched for in AML patients by a reverse transcription polymerase chain reaction (RT‐PCR). Based on these laboratory findings, the patient was diagnosed with de novo MS.

Initially, we treated the patient with local irradiation to the vertebral tumor, which immediately resolved the neurological symptoms. Additionally, we treated the patient with daunorubicin and cytarabine, followed by a course of high‐dose cytarabine. At the end of chemotherapy, the PET/CT showed no abnormal uptake.

Four months later, the MS relapsed as multiple tumors involving the right ribs. Because the tumors were localized, we attempted radiation therapy again. However, this time, the tumor was resistant to irradiation and soon expanded to multiple systemic bone tumors. We reevaluated the bone marrow, but leukemic cells were not detected morphologically and cytogenetically. Salvage chemotherapy with mitoxantrone and high‐dose cytarabine followed by a subsequent intrathecal injection of methotrexate was performed and resulted in a second remission. We recommended allogenic stem‐cell transplantation as a consolidation therapy, but the patient refused transplantation.

**Figure 3:**
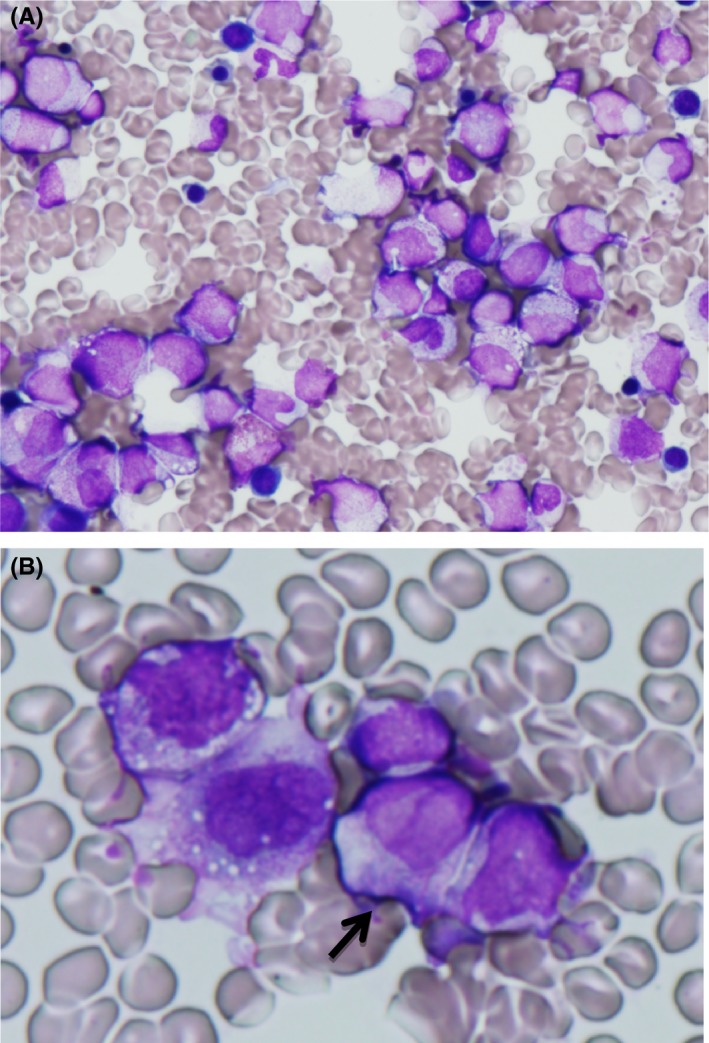
Bone‐marrow smear of second relapse phase (May‐Grunwald Giemsa stain, original magnification ×400). A, Proliferation of aberrant promyelocytes. B, The faggot cell (arrow, ×1000).

The second remission lasted for 6 months after the termination of the treatment. This time, the patient relapsed concomitant with leukopenia, thrombocytopenia, and disseminated intravascular coagulation (DIC). The bone marrow contained aberrant promyelocytes and faggot cells (Figure 3). The PML/RARα fusion gene was detected in 49% of cells by FISH, and also by RT‐PCR.

Finally, the diagnosis of APL was made. Chromosomal analysis showed a complex karyotype (47, XY, +8, der(11;22)(q10;q10), add(14)(q32), der(15)*t*(15;17)(q22;q12), ider(17)(q10)*t*(15;17)). At this point, we re‐examined the initial sarcoma sample, which was paraffin embedded and stored. We were able to detect the fusion signal of PML/RARα using FISH in the preserved sample and concluded it was de novo MS/APL from the onset of the disease.

We treated the patient with a combination of all‐trans retinoic acid (ATRA), daunorubicin, and cytarabine, which is the standard induction therapy for APL patients in our institute. Differential syndrome did not occur during treatment with ATRA. Hematological remission was acquired 39 days afterward, yet the PML/RARα fusion gene was still detected in bone marrow by RT‐PCR. Although we subsequently treated the patient with a combination of arsenic trioxide (ATO) and ATRA, the copy number of the PML/RARα fusion gene started to increase 9 weeks after starting ATRA therapy. Hematological recurrence became prominent 4 weeks after. Salvage treatment with gemtuzumab ozogamicin and tamibarotene was not sufficient for achieving remission. The patient died of a brain hemorrhage due to DIC induced by refractory APL shortly afterward, a total of 40 months from onset.

## 3 DISCUSSION

Within leukemic APL patients, MS/EMD mostly occurs in the relapse phase. The most common sites of involvement are the skin and central nerve system (CNS). High white blood cell count (WBC) and younger age are suggested as risk factors.^2^, ^3^, ^4^ Although the mechanisms of EMD are not clearly understood, this may result from extended survival induced by gene‐targeting ATRA and ATO therapies. In addition, ATRA distributes to the CNS only at low concentrations and may explain the high frequency of CNS involvement.^5^ An ex vivo experiment showed that ATRA increases adhesion molecules in leukemic cells, indicating the possibility of enhancing migration and adhesion to extramedullary tissues.^6^ However, a large cohort study demonstrated no increased risk of developing EMD for ATRA‐based therapy compared to chemotherapy alone.^7^ Because EMD usually occurs in the relapse phase and many CNS cases are included, survival after developing EMD is poor. The combination of intrathecal injection and chemotherapy is often chosen as the initial salvage treatment. Subsequent autologous or allogeneic stem‐cell transplantations are attempted as consolidation therapy, but the efficacy is not immediately evident.

On the other hand, de novo MS/APL is a rare condition, and clinical features may be distinctly different from EMD. We summarized 24 cases of MS as the first manifestation of APL found in the literature (Table 1). Nine cases were without bone‐marrow disease at the onset. Besides the three cases initially treated with ATRA, the remainders developed bone‐marrow involvement within 1‐16 months. The location of de novo MS was widely distributed, with many cases originating from the bone, especially from the spine. Nine cases showed neural symptoms because of MS compressing the spinal cord, which is different from the pattern of CNS invasion in EMD. Increased WBC was only seen in five cases. In addition, the coagulation abnormality characteristic of APL was evident only in five cases, and fifteen were presented with bone‐marrow disease.

**Table 1:** Published cases of de novo MS/APL

Case	Age/sex	Site of MS	BM involvement	Coagulation abnormality	WBC	ATRA therapy	Response (survival)	Ref
1	34/m	Skin	Yes	Yes	High	No	NR (1 mo)	11
2	4/m	Pelvis	Yes	No	High	No	CR (14 mo<)	12
3	23/m	Mediastine	No	No	Normal	No	NR (14 mo)	13
4	31/m	Extradura	No	Yes	Normal	No	PR (18 mo<)	14
5	21/m	Thymus	Yes	No	High	No	CR (8 mo)	15
6	27/m	Epidura	Yes	Yes	Normal	Yes	PR (13 mo<)	16
7	–/m	Skull, pleura, hip	Yes	–	Normal	Yes	CR (13 mo<)	17
8	66/m	Small intestine	Yes	No	Normal	No	Early death	18
9	55/m	Vertebra, epidura	No	No	Normal	Yes	CR (13 mo<)	19
10	18/m	Epidura	No	No	Normal	No	CR (10 mo<)	20
11	27/m	Testicle	No	No	Normal	Yes	PR (16 mo<)	21
12	39/f	Cerebellum	Yes	Yes	High	No	Early death	22
13	16/f	Humerus, tibia, femur	Yes	No	Normal	Yes	CR	23
14	45/m	Tongue	Yes	–	High	Yes	CR	24
15	26/f	Ovary	No	No	Normal	Yes^a^	CR (44 mo<)	25
16	17/f	Rectum	Yes	–	Normal	Yes	CR (4 y<)	26
17	19/m	Sternum	No	No	Normal	Yes	–	27
18	53/m	Extradura	Yes	Yes	Normal	Yes	HCR (early death)	28
19	26/m	Vertebra, Extradura	Yes	No	Low	Yes	CR (8 mo<)	29
20	29/m	Colon	Yes	No	Low	Yes	CR	30
21	1/m	Mandible	Yes	No	–	Yes	CR (1 y<)	31
22	61/f	Vertebra	No	–	–	Yes^a^	CR (8 y<)	32
23	52/f	Vertebra	No	No	Normal	Yes	CR (4.5 y<)	33
24	56/m	Vertebra	Yes	No	Normal	Yes	CR (15 mo<)	34
25	50/m	Vertebra	No	No	Normal	Yes^a^	CR (40 mo)	Present case

APL, acute promyelocytic leukemia; ATRA, all trans retinoic acid; BM, bone marrow; CR, complete response; HCR, hematological response; mo, month; MS, myeloid sarcoma; NR, no response; Ref, reference; WBC, white blood cell count; y, year.

a After radiation or chemotherapy.

As cases are reported independently, the optimum therapy is also unclear. Generally, AML‐type therapy is effective for de novo MS, resulting in compatible survival rates compared to the cytogenetic counterpart AML.^8^, ^9^ Sixteen de novo MS/APL cases were treated by ATRA with or without chemotherapy with acceptable responses, which is the standard therapy for leukemic APL (Table 1). On the other hand, for the eight cases treated without ATRA or ATO, only three were alive at the time of the report. In the present case, we started to treat the patient with gene‐targeting agents only after the second recurrence of the disease and could not achieve molecular remission. At that time, the karyotype of the bone‐marrow cells showed *t*(15;17) with a complicated abnormality and may have caused the resistance to treatment. It is interesting that differential syndrome was reported in five cases, including a case with isolated MS not involving bone marrow where a low tumor burden was predicted (case23).

Since de novo MS/APL is infrequently concomitant with coagulation abnormality or CNS disease, we conclude that optimal initial therapy with ATRA with or without chemotherapy may have a decent outcome. Therefore, molecular and cytogenetic information leading to accurate diagnosis is essential at the disease’s onset. Information may be obtained from the bone marrow in some cases, but 40% of MS/APL cases lack bone‐marrow disease, and thus, examination of the MS tissue becomes critical. When fresh tissue samples are not available, FISH can be performed on fixed and paraffin‐embedded sections to detect cytogenetic aberrations.^10^ Once the PML/RARα fusion gene is detected in a de novo MS, ATRA‐based therapy is recommended.

## CONFLICT OF INTEREST

None declared.

## AUTHOR CONTRIBUTION

TY: was the physician in charge of the patient and also prepared the manuscript. AN: was the member of the treatment team. YN: was the member of the treatment team. KN: was the member of the treatment team. GO: was the hematologist responsible for the treatment team. HT: is the hematologist responsible for this manuscript.
